# Non-convulsive status epilepticus possibly induced by a rapid correction of severe hyperkalemia: a case report and literature review

**DOI:** 10.1186/s12882-023-03141-1

**Published:** 2023-04-10

**Authors:** Saki Bussaka, Takaichi Suehiro, Koji Mitsuiki, Takato Morioka, Tadahisa Shono, Fujio Fujiki, Toshiaki Nakano

**Affiliations:** 1 Department of Nephrology, Harasanshin Hospital, 1-8, Taihakumachi, Hakata-Ku, Fukuoka, Fukuoka 812-0033 Japan; 2 Department of Neurosurgery, Harasanshin Hospital, Fukuoka, Japan; 3 Department of Neurology, Harasanshin Hospital, Fukuoka, Japan; 4 Department of Medicine and Clinical Science, Graduate School of Medical Sciences, Kyushu University, Fukuoka, Japan

**Keywords:** Non-convulsive status epilepticus, Hyperkalemia, Hemodialysis, Chronic kidney disease, Consciousness disturbance

## Abstract

**Background:**

Patients with chronic kidney disease frequently develop neurological complications including confusion and altered consciousness. Non-convulsive status epilepticus, which is characterized by a change in behavior and/or mental process accompanied by epileptiform discharges on electroencephalogram in the absence of convulsive seizures, is one of the overlooked causes of altered consciousness. The incidence and precise pathophysiological mechanism of non-convulsive status epilepticus in patients with kidney disease, and especially in patients with electrolyte disturbances, remains unknown. We recently treated an older patient with chronic kidney disease and severe hyperkalemia in whom non-convulsive status epilepticus developed following a correction of severe hyperkalemia.

**Case presentation:**

An 82-year-old male was admitted to our hospital at midnight because of weakness of all four limbs (Day 1). He underwent urgent hemodialysis for severe hyperkalemia (9.84 mEq/L) and his serum potassium concentration decreased to 4.97 mEq/L. He regained full consciousness and his limb weakness improved on the morning of Day 2, but he became confused in the evening. Electroencephalogram revealed repeated low-voltage ictal discharges in the right occipital region and a diagnosis of non-convulsive status epilepticus was made. Following medication with fosphenytoin and phenytoin, the patient became fully alert and orientated on Day 8.

**Conclusion:**

We speculate that a rapid correction of hyperkalemia was the possible cause of non-convulsive status epilepticus development. To our knowledge, this is the first report of non-convulsive status epilepticus from a potassium abnormality. We described a case of this condition in detail and summarized 78 previous case reports of non-convulsive status epilepticus with kidney disease or electrolyte disturbances.

## Background

Neurological complications including confusion and altered consciousness are commonly encountered in chronic kidney disease (CKD) patients. Altered consciousness in patients with CKD is caused by non-convulsive status epilepticus (NCSE), uremic encephalopathy, disequilibrium syndrome, dialysis dementia, infection, drugs, electrolyte imbalances, hypoxia, hypertensive crisis, or cerebrovascular disease [1–4].

NCSE is generally defined as a change from the baseline in behavior and/or mental process that is associated with ongoing epileptic activities or continuous epileptiform discharges on electroencephalogram (EEG) in the absence of convulsive symptoms [5, 6]. Prompt diagnosis of NCSE is important because this condition is potentially reversible with appropriate treatment; however, NCSE is often misdiagnosed as a cause of an acute state of confusion when EEG is not used [1–4]. Because there are few reports of NCSE in patients with CKD [1–4], the precise pathophysiological mechanism of NCSE development with kidney disease remains unknown.

We recently treated an older patient with CKD in whom NCSE was thought to be induced by a rapid correction of severe hyperkalemia using sodium bicarbonate, glucose–insulin (GI) therapy, and hemodialysis (HD).

## Case presentation

An 82-year-old man developed diarrhea and abdominal pain starting at noon (Day 1). He had stage 4 CKD of unknown etiology and no history of epilepsy except for febrile seizure in childhood. In the evening of Day 1, weakness in all four limbs occurred, and he was admitted to our hospital by ambulance.

Vitals were temperature 36.3 °C, blood pressure 163/69 mmHg, and pulse oximetry 100%. Arterial blood gas test results revealed hyperkalemia (9.84 mEq/L) and metabolic acidosis (pH 7.227, PCO_2_ 25.6 mmHg, PO_2_ 143.7 mmHg, and HCO_3−_ 10.5 mEq/L). Blood tests revealed blood urea nitrogen was 93.7 mg/dL, creatinine 4.77 mg/dL, blood glucose 154 mg/dL, ammonia 52 µg/dL, sodium 133.6 mEq/L, corrected calcium 9.2 mg/dL, and magnesium 1.7 mg/dL. Electrocardiogram results were characteristic of hyperkalemia including a tentorial T wave, prolonged QT, wide QRS, and irregularity in R-R. In the outpatient clinic, his serum potassium was controlled between 4.35–5.15 mEq/L with oral calcium polystyrene sulfonate; however, his family doctor changed 30 mg of azosemide, which was used to treat chronic heart failure, to 25 mg of spironolactone 2 weeks earlier. He had also been eating a large amount of fruit including apples and ponkan oranges over the previous week.

**Fig. 1:**
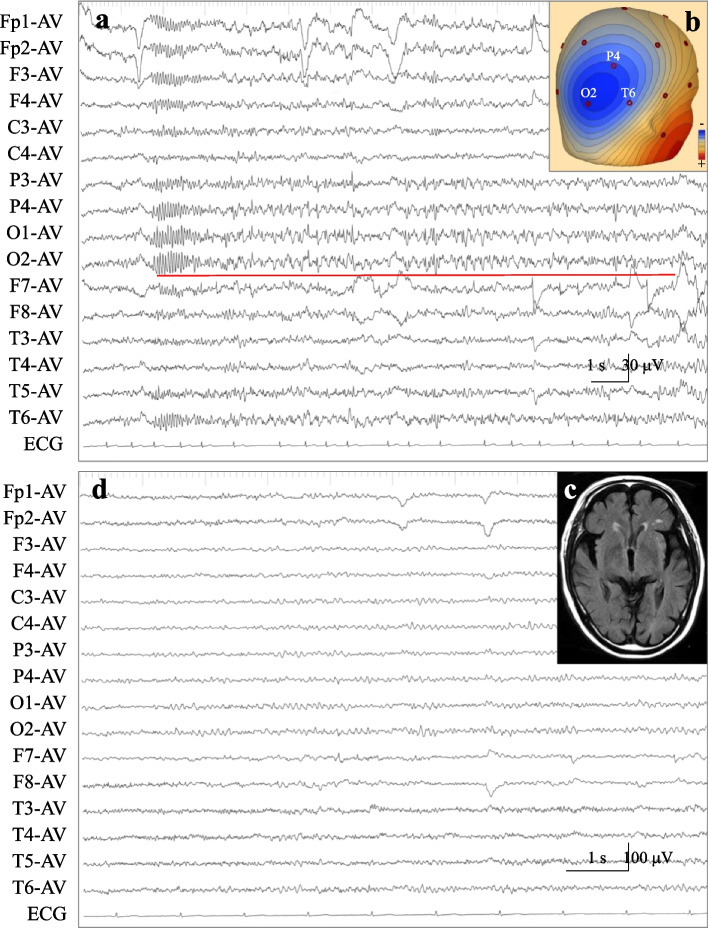
Electroencephalogram, voltage topography and magnetic resonance imaging findings. **a** An electroencephalogram (EEG) on Day 5 showing repeated low-voltage ictal discharges from the right occipital region (red line). Note that the sensitivity of EEG recording is displayed at approximately three-times higher than the ordinary sensitivity (AV, averaged reference). Atrial fibrillation was also observed on electrocardiogram (ECG). **b** Voltage topography demonstrates the negativity of the ictal discharges to be located at O2, P4, and T6 of the International 10–20 EEG system. **c** A subsequent magnetic resonance image with fluid-attenuated inversion recovery sequence showed no epileptogenic lesion or abnormal edema. **d** EEG on Day 11 depicting the disappearance of paroxysmal discharges. The dominant rhythm of the α-ranged wave can be observed on both sides

After sodium bicarbonate administration and GI therapy, urgent HD was performed for 2 h with a blood flow rate of 120 mL/min and dialysate flow rate of 500 mL/min using a 0.8m^2^ small surface area dialyzer (APS-08SA, Asahi Kasei Medical Co., Tokyo, Japan). Dialysate sodium was 140 mEq/L, potassium 2.0 mEq/L, and bicarbonate 27.5 mEq/L. After dialysis, venous blood gas test results revealed serum potassium 4.97 mEq/L, pH 7.394, PCO_2_ 34.7 mmHg, PO_2_ 28.2 mmHg, and HCO_3−_ 20.8 mEq/L. Blood tests revealed blood urea nitrogen 59.4 mg/dL, creatinine 3.32 mg/dL, blood glucose 95 mg/dL, sodium 137.6 mEq/L, corrected calcium 9.9 mg/dL, and magnesium 1.5 mg/dL. After this HD session, his serum potassium was controlled between 4.18 and 5.39 mEq/L. His verbal responses became accurate and limb weakness improved. Nevertheless, he became slow to react to external stimuli after 6 h, and after 17 h he became confused and irritable, which was uncontrollable with sedatives including haloperidol and quetiapine. On Day 5, EEG with increased sensitivity (to three times the ordinary conditions) demonstrated low-voltage ictal discharges with evolution in frequency and morphology lasting more than 10 s in the right occipital region (Fig. 1a), with maximal amplitudes in O2, P4, and T6 using the International 10–20 EEG system (Fig. 1b). The ictal discharges were observed for approximately 25% of the 60-min recording period, and a diagnosis of electrographic status epilepticus was made based on the American Clinical Neurophysiology Society’s Standardized Critical Care EEG Terminology [7]. No epileptogenic lesions or abnormal edema were noted on subsequent magnetic resonance images (Fig. 1c).

With a clinical diagnosis of focal NCSE based on the International League Against Epilepsy (ILAE) classification [6], 750 mg of fosphenytoin was administered intravenously on Day 5, followed by an additional 375 mg of fosphenytoin on Day 6. Phenytoin 200 mg was also administered orally. His mental state gradually improved, and he became fully alert and orientated on Day 8. EEG on Day 11 showed that the epileptic discharges had disappeared (Fig. 1d). Oral phenytoin administration was discontinued. He was ambulatory when discharged and returned to normal daily life.

## Review of the literature

### Methods

We searched case reports related to our research published in English, and manually revised the reference lists of relevant articles. We also searched reviews to identify any papers that were missed by our search strategy.

We searched the PubMed database using a combination of Medical Subject Headings (MeSH) terms and keywords related to NSCE, renal dysfunction, and electrolyte disturbances, as follows: non-convulsive status epilepticus, “Status Epilepticus” [MeSH], renal dysfunction, kidney injury, renal failure, “Kidney Diseases” [MeSH], hemodialysis, “Renal Dialysis” [MeSH], hyperkalemia, hypokalemia, hypernatremia, hyponatremia, hypercalcemia, hypocalcemia, hypermagnesemia, hypomagnesemia, and “Water-Electrolyte Imbalance” [MeSH]. Patients older than 19 years were included in the search.

Two authors (SB and TSu) independently evaluated the articles for eligibility by first screening the title and abstract, and then the full text. The search was conducted in June 2021, and there were no time limitations for study inclusion.

## Results

The NCSE cases in patients with electrolyte disturbance are listed in Table 1a [8–13]. NCSE occurred during hyponatremia, hypocalcemia, hypokalemia, and hypomagnesemia, as well as after correction of hypercalcemia and hyperkalemia.

**Table 1:** Summary of reported cases with nonconvulsive status epilepticus with renal dysfunction or electrolyte disorder excluding cefepime cases

Patient no	Age (years)/gender	Diagnosis	Creatinine (mg/dL)	Renal function	Dialysis mode	Pathogenesis of NCSE	Clinical signs	Treatment	Outcome	Author	Reference
a. Electrolyte disturbances											
1	46/M	Idiopathic hypoparathyroidism	NR	Normal	-	Hypocalcemia (Ca 4.1 mg/dL) (stopped using supplemental calcium)	Brief, tonic–clonic seizure, restless, unable to speak subsequent	Lorazepam, 10% calcium chloride	Became verbal and fully oriented	Kline	[10]
2	77/F	Total thyroidectomy, hypercalcemia	2.3	AKI	-	Rapid correction of hypercalcemia (14.4 mg/dL to 8.8 mg/dL)	Confused, agitated, disorientated, visual and auditory hallucinations	Phenytoin, carbamazepine	Improved continuously becoming more communicative and less agitated	Kümpfel	[8]
3	57/M	Primary insomnia, polydipsia induced hyponatremia	NR	Normal	-	Hyponatremia (118 mEq/L)	Poor orientation, memory disturbances, a decrease in spontaneous speech, bradykinesia, confusion, consciousness disturbance	Saline infusion, diazepam, phenytoin	Improved EEG and conscious disturbance	Azuma	[11]
4	56/M	Syndrome of inappropriate ADH production	NR	Normal	-	Hyponatremia (121 mEq/L)	Confusion, vague and distractible, persistent hypertonia, and a new waxy catatonia	0.9% saline infusion, tolvaptan, midazolam, phenytoin	Abolished abnormal electrical activity, communicate normally	Lovell	[12]
5	53/F	Borderline personality disorder, water intoxication	NR	Normal	-	Hyponatremia (90 mEq/L)	Generalized tonic–clonic seizures, followed by mental confusion	Electrolyte infusion (NaCl), water restriction, lorazepam, phenobarbital	Mental status and EEG were restored to normal	Primavera	[13]
6	67/F	Diarrhea, hypokalemia (2.8 mEq/L), hypomagnesemia (0.4 mg/dL)	NR	NR	-	NR	Confused and developed generalized motor and non-motor seizures	Replacement of K + and Mg2 + , oxcarbazepine, levetiracetam, sodium valproate, phenytoin	Resolution of the neurological states	Binaghi	[9]
7	82/M	Hyperkalemia, prostate cancer, hypertension, chronic heart failure, febrile seizure	4.8	CKD	Temporary HD	Rapid correction of hyperkalemia (9.84 mEq/L to 4.97 mEq/L)	Became confused and irritable	sodium bicarbonate infusion, GI therapy, fosphenytoin, phenytoin	Mental state gradually improved, EEG depicted the disappearance of epileptic discharges	Our case	
b. Renal dysfunction excluding the case of cefepime											
1	40/F	Pneumonia, diabetes mellitus, post transplantation lymphoproliferative disorder	11.9	CKD	PD	Ceftazidime	Stupor	Lorazepam, phenytoin, phenobarbitone	Improved EEG appearance mainly	Chow	[1]
2	72/F	Peritonitis	4.8	CKD	PD	Ceftazidime	Mute, mild extrapyramidal signs, sporadic myoclonic jerks	Lorazepam	Showed a complete recovery of mental status and neurological findings	Primavera	[14]
3	64/M	Pneumonia	8.0	CKD	-	Ceftazidime	Agitation, confusion, myoclonus	Clonazepam, phenytoin	Improved clinical symptoms	Martinez-Rodriguez	[15]
4	38/M	Pneumonia	6.7	CKD	-	Ceftazidime	Confusion, myoclonus	Clonazepam	Improved clinical symptoms	Martinez-Rodriguez	[15]
5	70/F	Peritoneal dialysis-related peritonitis	3	CKD	PD	Ceftazidime	Mutism, asterixis, and horizontal nystagmus	Diazepam	Full recovery	Vannaprasaht	[16]
6	46/F	Pseudomonas aeruginosa peritonitis	12.5	CKD	PD	Ceftazidime, ciprofloxacin	Confusion	Diazepam, phenytoin	Seizure activity under control on EEG and improved mental state, but died of nosocomial pneumonia	Chow	[1]
7	67/F	Febrile neutropenia, multiple myeloma	4.8	CKD	Temporary HD	Ceftazidime, ciprofloxacin	Stupor, rare limb contractions	Diazepam, clonazepam	Died of cardiopulmonary arrest	Ozturk	[17]
8	65/F	Urinary tract infection	6.7	CKD	Temporary HD	Ceftriaxone	Stupor, generalized myoclonic jerks	Discontinue of ceftriaxone	Improve neurologic signs or symptoms	Kim	[18]
9	71/M	Urinary tract infection, hepatic carcinoma	5.3	CKD	-	Ceftriaxone	Meaningless speech, an inability to walk, sleepiness	Diazepam	Improved clinical symptoms and EEG findings	Bora	[19]
10	78/F	Meningitis	5.2	CKD	-	Ceftriaxone	Drowsiness, myoclonus	Valproate	Improved clinical symptoms	Martinez-Rodriguez	[15]
11	24/F	Urinary tract infection, lupus nephritis after renal transplantation, hypertension	2.8	CKD	-	Ceftriaxone	Confusion after generalized tonic–clonic seizures	Diazepam, phenytoin	Clinical symptoms and EEG abnormalities improved	Bora	[19]
12	83/F	Pneumonia	2.0	AKI	-	Ceftriaxone	Drowsiness, myoclonus	Phenytoin, valproate	Improved clinical symptoms	Martinez-Rodriguez	[15]
13	55/F	Acute pyelonephritis, polycystic kidney	3.7	AKI	-	Cefoperazone, sulbactam	Generalized convulsion	Diazepam, phenytoin	Resolved	Ozturk	[17]
14	52/M	Pneumonia, transplanted kidney, transplant renal artery stenosis	2.8	CKD	-	Imipenem, cilastatin	Contractions of face muscles, stupor	Phenytoin	Died of heart failure	Ozturk	[17]
15	58/F	Tuberculosis, diabetes mellitus	15.0	CKD	PD	Isoniazid, rifampicin	Confusion	Lorazepam, phenytoin	Full recovery	Chow	[1]
16	72/M	Diabetes mellitus	7.1	CKD	HD	Star fruit poisoning	Comatose, nystagmus, right hemiplegia	Phenytoin	Consciousness became clear and walk independently	Chang	[20]
17	65/M	Convulsive generalized epilepsy	NR	CKD	HD	Drug levels of antiepileptic medications (phenobarbital)	Confused, irritable	Diazepam, phenobarbital	No more epileptic seizures or NCSE appeared	Marinelli	[4]
18	62/M	Diabetes mellitus, hypertension, old ischemic stroke	9.9	CKD	HD	Drug levels of antiepileptic medications (carbamazepine)	Confusion, abnormal eye movements	Diazepam, carbamazepine	Improved the conscious level, follow-up EEG revealed no abnormal	Iftikhar	[2]
19	64/F	Diabetes mellitus, hypertension, atrial fibrillation, old stroke	9.2	CKD	-	Drug levels of antiepileptic medications (phenytoin)	Confusion, minor regular twitches of eyes and lips	Diazepam, phenytoin	Subsequent EEG was normal	Iftikhar	[2]
20	75/F	Chronic kidney disease	4.5	CKD	HD	NR	Confusion, abnormal eye movements	Diazepam	Got good electrical and clinical responses, and follow-up EEG was normal	Iftikhar	[2]
21	41/M	Pancreatitis, human immunodeficiency virus	NR	CKD	NR	Systemic metabolic disorder and HIV related encephalopathy	Memory and cognitive loss, confusion, incoherent	Benzodiazepine, phenobarbital	EEG showed no recurrence of seizure activity, but persisted moderate memory and cognitive problems	Krumholz	[21]
22	56/F	Hemolytic uremic syndrome	NR	AKI	Temporary HD	Hemolytic uremic syndrome	Focal seizures	Midazolam	No seizure recurrence	Braksick	[22]

*NCSE* non-convulsive status epilepticus, *CKD* chronic kidney disease, *PD* peritoneal dialysis, *EEG* electroencephalogram, *HD* hemodialysis, *AKI* acute kidney injury, *NR* not reported, *HIV* human immunodeficiency virus, *ADH* antidiuretic hormone, *GI* glucose-insulin, *F* female, *M* male

The NCSE cases in patients with renal dysfunction are listed in Tables 1b and 2. Twenty-two NCSE cases, excluding cefepime-related NCSE, are listed in Table 1b [1, 2, 4, 14–22]. Fifteen cases were reported as antibiotic-related NCSE. Among the causes unrelated to antimicrobial agents, abnormal blood levels of antiepileptic drugs were reported in three patients. Human-immunodeficiency-virus-related encephalopathy, hemolytic uremic syndrome, and neurotoxicity caused by star fruit were each reported in one case only. Most patients improved well with treatment. Table 2 lists 49 cases of cefepime-related NCSE in patients with renal dysfunction [1–3, 14, 15, 17, 19, 20, 23–38].

**Table 2:** Summary of reported cases with nonconvulsive status epilepticus with renal dysfunction and cefepime

Patient no	Age / gender	Diagnoses	Creatinine (mg/dL)	Renal function	Dialysis mode	Clinical signs	Treatment	Outcome	Author	Reference
1	61/F	Febrile neutropenia, multiple myeloma	8.0	CKD	HD	Contractions of eyelids, chin, hands, and arms	Diazepam, clonazepam	Improved clinical symptoms	Ozturk	[17]
2	72/M	Central line sepsis, hepatitis B, diabetes mellitus, hypertension, seizure disorder, moderate obesity	6.8	CKD	HD	Confusion	Diazepam	Condition recovered gradually; EEG study showed no abnormal discharges	Iftikhar	[2]
3	44/M	Pneumonia, bilateral orthotopic lung transplant	5.8	CKD	HD	Confusion, obtundation, clonus	Lorazepam, valproic acid	Rapid recovery in mental status, absent epileptiform discharges in EEG, although showed mild slowing of the background	Dixit	[23]
4	52/M	Urinary tract infection, hypertension, diabetes mellitus	3.4	CKD	HD	Delirium, disorientation, agitation, myoclonic jerks	Diazepam	Clinical symptoms and EEG findings improved	Bora	[19]
5	68/F	Pneumonia, diabetes mellitus	NR	CKD	HD	Confusion, stupor, lip smacking	Valproic acid, lorazepam	EEG findings and her mental state improved gradually	Lee	[3]
6	29/M	Brain abscess	2.4	CKD	HD	Stupor	Diazepam, phenytoin	NR	Ozturk	[17]
7	NR	NR	NR	CKD	HD	Confusion with global aphasia	Discontinue of cefepime	Neurologic and electroencephalographic status normalized in a few days	Barbey	[37]
8	69/F	Pneumonia	14.2	CKD	PD	Confusion	Diazepam, phenytoin	EEG improvement followed by mental recovery	Chow	[1]
9	48/F	Amyloidosis susp, bronchiectasis	7.5	CKD	PD	Agitation, speech difficulty, hand tremor, loss of consciousness	Diazepam	Consciousness improved	Ozturk	[17]
10	48/F	Acute bronchitis, hypertension	5.2	CKD	PD, temporary HD	Confusion	Diazepam, phenytoin	Made a good recovery	Iftikhar	[2]
11	49/F	Kidney transplant, acute pyelonephritis	CCr 39 ml/min	CKD	-	Stupor	Lorazepam, levetiracetam	Epileptiform activities ceased and regained her baseline mental status	Balderia	[38]
12	39/F	Septic arthritis, ankylosing spondylitis, amyloidosis	6.5	CKD	-	Agitation, confusion, myoclonic jerks	Diazepam	Clinical symptoms and EEG findings improved	Bora	[19]
13	83/F	Pneumonia, lung tuberculosis, chronic atrial fibrillation	6.1	CKD	-	Stupor	Diazepam, phenytoin	Consciousness improved completely	Ozturk	[17]
14	33/F	Febrile neutropenia, cellulitis, chronic allograft nephropathy, diabetes mellitus	6.0	CKD	-	Disorientation, loss of consciousness	Diazepam, phenytoin	Improved clinical symptoms	Ozturk	[17]
15	86/M	Osteomyelitis	5.1	CKD	-	Agitation, confusion, myoclonus	Valproate, phenytoin	Improvement of clinical symptoms	Martinez-Rodriguez	[15]
16	64/F	Pneumonia	5.0	CKD	-	Agitation, confusion, myoclonus	Clonazepam, phenytoin	Clinical improvement immediately	Martinez-Rodriguez	[15]
17	58/F	Bronchopneumonia, mesothelioma, diabetes mellitus, coronary artery disease	4.7	CKD	-	Less responsive, unable to speak	Diazepam	Clinical and neurophysiological findings began to normalize	Bora	[19]
18	79/M	Pneumonia	4.5	CKD	-	Confusion, myoclonus	Clonazepam, valproate	Improvement of clinical symptoms	Martinez-Rodriguez	[15]
19	68/M	Pneumonia, osteomyelitis, prostate cancer, bone metastases	4.0	CKD	-	Confusion, disorientation	Diazepam	Remission of neuropsychological and clinical findings	Bora	[19]
20	67/F	Pneumonia	3.5	CKD	-	Drowsiness, confusion	Clonazepam	Clinical improvement immediately	Martinez-Rodriguez	[15]
21	54/M	Liver cirrhosis, prophylaxis	3.2	CKD	-	Agitation, confusion	Clonazepam, diazepam	Improvement of clinical symptoms	Martinez-Rodriguez	[15]
22	70/F	Febrile neutropenia, non-Hodgkin’s lymphoma, hypertension, seizure disorder,	2.8	CKD	-	Agitation, myoclonus	Levetiracetam, phenytoin	Returned to baseline mental function	Gangireddy	[24]
23	86/M	Right lower lobe pneumonia	2.5	CKD	-	Agitation, confusion	Lorazepam, phenytoin	EEG showed resolution of the epileptiform activity, remained alert and conscious	Chang	[20]
24	58/F	Urinary infection, acute rejection, pyelonephritis, transplanted kidney	2.0	CKD	-	Impaired consciousness	Diazepam	Consciousness improved immediately	Ozturk	[17]
25	50/F	Pneumonia, lung transplant, chronic myeloid leukemia	2.0	CKD	-	Nonverbal, unable to follow commands, myoclonic jerking	Lorazepam, levetiracetam	Mental status was normalized	Tchapyjnikov	[25]
26	28/F	Urinary tract infection, thoracic spina bifida, hydrocephalus (after ventriculoperitoneal shunt)	1.8	CKD	-	Confusion, twitching movements of both upper extremities	Lorazepam, valproic acid	Returned to her baseline health, EEGs did not reveal epileptiform discharges or electrographic seizures	Dixit	[23]
27	57/F	Heart failure, diabetes, ischemic cardiomyopathy	1.7	CKD	-	Loss of orientation, diminished speaking, difficulty following commands, visual hallucinations	Lorazepam	Mental status returned to baseline; EEG showed resolution of triphasic discharges	Tchapyjnikov	[25]
28	70/F	Pneumonia, lung transplant, a-1 antitrypsin deficiency	1.7	CKD	-	Disorientation, nonverbal, inability to follow commands	Discontinuation of cefepime only	Mental status normalized with associated resolution of triphasic wave discharges on EEG	Tchapyjnikov	[25]
29	72/F	Osteomyelitis, hypertension, hyperlipidemia, asthma, peripheral vascular disease, past breast cancer	1.6	CKD	-	Agitation, somnolence	Benzodiazepines	Mental status returned to normal	Lichaa	[26]
30	36/M	Urinary tract infection, nephrotic syndrome, past intracerebral hemorrhage	CCr 49 ml/min	CKD	-	Global aphasia, motor aphasia	Discontinuation of cefepime only	EEG and clinical symptoms made a gradual recovery	Kwon	[27]
31	60/M	Urinary tract infection, prostatic/hepatic abscess, critical illness polyneuropathy, pneumonia, after liver transplant	1.8	CKD	-	Reduction level of consciousness, myoclonic jerks	Midazolam	EEG confirmed moderate diffuse encephalopathy without epileptiform activity, died of severe heart failure	Fernández-Torre	[33]
32	79/F	Pneumonia	5.2	CKD	-	Confusion, myoclonus	Clonazepam, phenytoin, valproate	EEG and clinical improvement but died of heart failure	Martinez-Rodriguez	[15]
33	85/F	Pneumonia, late hemorrhagic cerebrovascular accident, diabetes mellitus, hypertension	1.5	CKD	-	Stupor	Diazepam	Status presentation and mental state did not improve	Ozturk	[17]
34	60/F	Ventilator-associated pneumonia, febrile neutropenia, myelodysplasia	CCr 12 ml/min	CKD	-	Facial convulsions	Valproic acid, phenytoin, levetiracetam	Died of intractable invasive aspergillosis	Spriet	[28]
35	54/M	Neutropenic fever, myelodysplasia	CCr 13 ml/min	CKD	-	NR	Valproic acid, phenytoin, phenobarbital	Neurologic status did not improve, died of septic shock	Spriet	[28]
36	74/M	Bronchopneumonia	10.1	AKI	Temporary HD	Stupor	Diazepam	Complete recovery of mental status, disappearance of paroxysmal activity on EEG paroxysmal activity on EEG	Primavera	[14]
37	40/F	Pyelonephritis, upper gastrointestinal bleed, acute hypoxic respiratory failure	5.1	AKI	Temporary HD	Twitching face and bilateral upper extremities	Lorazepam, levetiracetam, midazolam,	Twitching stopped and no further electrographic seizures or triphasic waves were seen on EEG	Oyenuga	[29]
38	65/M	Mediastinitis, esophageal carcinoma	3.2	AKI	Temporary HD	Confusion, myoclonus	Clonazepam, antiepileptic drugs	Complete recovery	Chatellier	[30]
39	71/F	Atypical pneumonia, lung cancer (after right lower lobe lobectomy), diabetes mellitus, hepatitis B viral infection	1.3	AKI	Temporary HD	Confusion, disorientation, stupor, generalized myoclonic seizure	Lorazepam, levetiracetam, valproic acid, phenytoin, clonazepam	EEG and clinical symptoms improved	Kim	[31]
40	65/F	Urinary tract infection, severe nephrolithiasis	CCr 20 ml/min	AKI	Temporary HD	Disorientation, dysarthria, myoclonus	Diazepam, valproic acid, levetiracetam	Regain previous mental condition, EEG perform normal	Suarez-de-la-rica	[32]
41	38/F	Neutropenia, allogenic bone marrow transplantation, hypernephroma	3.2	AKI	-	Agitation, disorientation	Diazepam, phenytoin, benzodiazepines	Completely controlled	Fernandez-Torre	[33]
42	76/F	Gangrenous pyoderma, neutropenia, chronic alcoholism	3.0	AKI	-	Agitation, alteration of consciousness level	Diazepam, phenytoin	Completely the epileptiform activity, mental status was normal	Fernandez-Torre	[33]
43	65/M	Nodular sclerosing Hodgkin's disease	2.8	AKI	-	Decreased level of consciousness, myoclonic jerks	Phenytoin	Neurological examination was normal, but a third relapse of Hodgkin's disease occurred and subsequently died	Plensa	[34]
44	43/M	Abdominal sepsis, congenital megacolon, chronic alcoholism	1.7	AKI	-	Abnormal behavior, severe mutism	Phenytoin	Experienced a dramatic clinical improvement	Fernandez-Torre	[33]
45	72/F	Pelvic osteomyelitis, diabetes, hypertension, chronic indwelling Foley catheter, coronary artery disease	1.2	AKI	-	Acute aphasia, confusion	Lorazepam, levetiracetam	Regained verbal function, EEG showed bi-hemispheric slowing but with no further evidence of seizure	Cunningham	[35]
46	66/F	Acute myeloid leukemia, pancytopenia, hyperlipidemia	CCr 30 ml/min	AKI	-	Confusion, myoclonic jerk	Clonazepam, valproic acid	EEG performed absence of epileptiform activity, regained consciousness	Abanades	[36]
47	73/F	Infection of knee prosthesis, rheumatoid, arthrosis	2.8	AKI	Temporary HD	Coma	Clonazepam, antiepileptic drugs	Died of multi-organ failure	Chatellier	[30]
48	55/F	Febrile neutropenia, pancytopenia after chemotherapy, refractory lymphoma	5.1	AKI	-	Loss of consciousness	Diazepam	Consciousness did not improve and died of sepsis	Ozturk	[17]
49	75/F	Pneumonia, congestive heart failure	2.2	AKI	-	Confusion	Diazepam, phenytoin	Died of sepsis and congestive heart failure	Ozturk	[17]

*NCSE* non-convulsive status epilepticus, *CKD* chronic kidney disease, *HD* hemodialysis, *EEG* electroencephalogram, *NR* not reported, *PD* peritoneal dialysis, *AKI* acute kidney injury, *F* female; M, male

## Discussion and conclusions

The present case had no epileptogenic lesions, but he developed de novo focal NCSE in the right occipital region following a rapid correction of hyperkalemia. To our knowledge, this is the first case of NCSE associated with a potassium abnormality; however, the precise mechanism underlying this NCSE development remains unknown.

Causes of consciousness disorders in patients with CKD include NCSE, uremic encephalopathy, disequilibrium syndrome, dialysis dementia, infection, drugs, electrolyte imbalances, hypoxia, hypertensive crisis, and cerebrovascular disease [1–4]. Dialysis disequilibrium syndrome must first be ruled out when a patient experiences consciousness disorder after hemodialysis. Dialysis disequilibrium syndrome generally occurs in patients with severe azotemia undergoing high-efficiency hemodialysis. Our patient did not have severe azotemia or undergo high-efficiency hemodialysis. Our dialysis prescription was of lower efficiency than that suggested to prevent dialysis disequilibrium syndrome, which uses a low initial blood flow rate (150 to 250 mL/min) with a small surface area dialyzer (0.9 to 1.2 m^2^) for 1–2 h [39]. Furthermore, head magnetic resonance imaging of our patient did not show cerebral edema, which has been documented in a case series of dialysis disequilibrium syndrome [40]. Previous reports of EEG findings in dialysis disequilibrium syndrome show slow waves in background activities that indicate cerebral dysfunction [41, 42]; there are no published reports of electrographic status epilepticus such as that seen in the present case. Together, these findings suggest that dialysis disequilibrium syndrome is not consistent with our case. Uremic encephalopathy is another possible cause of consciousness disorder, but was unlikely in our patient. He did not have severe azotemia and was confused after hemodialysis. All other possible causes of consciousness disorder were also unlikely based on the clinical findings, medical history, laboratory data, and imaging findings of our patient. We therefore concluded that NCSE was the cause of consciousness disturbance in our patient.

The etiology of NCSE includes a wide variety of diseases [1–4, 8–38, 43, 44]. Thomas et al. [45] first reported de novo NCSE of frontal origin in patients with no epileptogenic lesion; this can be triggered by metabolic factors such as hyponatremia and non-ketotic hyperglycemia, drug withdrawal (especially from benzodiazepine), potentially epileptogenic drug prescription, or in many cases a combination of several of these factors. The NCSE clinical entity in the present case can be termed “situation-related NCSE” [5, 46]. From previous reports, the possible etiology of NCSE in our patient was electrolyte disorder or renal failure; other factors were absent in our patient before NCSE development. We have summarized the previously reported NCSE cases associated with renal dysfunction or electrolyte disorder in Tables 1a, b, and 2. Renal dysfunction or uremia can be a possible cause of NSCE. Nevertheless, in the 71 reported cases of NCSE with renal dysfunction in Tables 1b and 2, uremia itself was not reported as a cause of NCSE in any patients. Additionally, patients with renal dysfunction developed NCSE as the result of other factors (mainly antibiotics). Because there are many reports of NCSE with renal dysfunction, uremia might lower the threshold for NCSE, but there is no evidence that uremia itself can cause NCSE. Changes in urea and acidosis, which cause dialysis disequilibrium syndrome, were one possible etiology of NCSE in our patient. Nevertheless, he had no typical signs of dialysis disequilibrium syndrome; we therefore speculated that the changes in urea and acidosis did not strongly impact our patient. Given that there was no apparent known etiology of NCSE in our patient, we hypothesized that a rapid correction of hyperkalemia, which is an electrolyte disorder, might have been the possible cause of his NCSE.

The most notable event in our case was extremely severe hyperkalemia. Cases of NCSE or epilepsy complicated by potassium abnormalities have rarely been reported. Binaghi et al. [9] noted that a patient with hypokalemia and hypomagnesemia developed NCSE and Takotsubo syndrome. Furthermore, Fujimura et al. [47] summarized 185 cases of Gitelman syndrome (also known as familial hypokalemia-hypomagnesemia) in which 2.5% of patients were diagnosed with epilepsy; they suggested that Gitelman syndrome or hypokalemia increases sensitivity to convulsions. In NCSE cases accompanied by potassium or calcium abnormalities [8–10], NCSE generally occurs following a change in serum potassium or calcium levels, as was noted in our patient. For example, Kümpfel et al. [8] reported that a patient with hypercalcemia (14.4 mg/dL) developed NCSE after the correction of hypercalcemia to a normal level (8.8 mg/dL), and suggested that the rapid decrease in serum calcium concentrations might have triggered the NCSE. Calcium plays a role in the pathogenesis of epileptic discharges, and disturbances in calcium homeostasis influence neuronal excitability and may lead to hyperexcitability [48]. Given that both potassium and calcium are related to cell excitability, potassium abnormality might also trigger NCSE or epilepsy.

The relationship between epilepsy development and changes in serum potassium concentrations has not been fully investigated, although there have been some reports of potassium abnormalities associated with epilepsy development [9, 49–51]. For example, mutations in the *KCNQ2* [51] and *KCNJ10* [49] genes, which encode potassium channels, have been reported to cause epilepsy. Bockenhauer et al. [49] reported that KCNJ10 channels modulate resting membrane potentials in excitable cells and cause epilepsy if mutated. Voltage-gated potassium channels in the central nervous system are easily activated, and intracellular potassium flows out of cells to decrease the membrane potential, thereby stabilizing membrane depolarization and the repetitive firing of action potentials [50]. It has also been reported that elevated extracellular potassium levels are associated with epilepsy [52, 53]. For example, Fröhlich et al. [53] suggested that the duration, magnitude, and rate of change of extracellular potassium concentrations can result in a transition to an epileptic condition. Similarly, Curtis et al. [54] demonstrated that when extracellular potassium concentrations rise significantly above physiological levels, a depolarization block and sustained seizures occur. These reports support the idea that a potassium abnormality can trigger NCSE development. We therefore speculated that a rapid decrease in extracellular potassium with urgent HD, combined with an increase in intracellular potassium with sodium bicarbonate and GI therapy, impaired suppression mechanisms against excitatory activity in our case.

Severe hyperkalemia as observed in the present case is rare and has a high mortality rate [55, 56]. The odds ratio of death within 1 day of severe (≥ 6.0 mEq/L) hyperkalemia in CKD stage 4 is 11.6 compared with patients with normokalemia (< 5.5 mEq/L) and no CKD [55], and the 3-year incidence of death in patients with potassium ≥ 8 mEq/L is at least 80% [56]. Electrolyte disturbances such as hyponatremia, hypernatremia, hypocalcemia, hypomagnesemia, and alkalosis are all associated with seizures [57]. Unlike other electrolyte disturbances, potassium abnormality rarely causes symptoms in the central nervous system, and there are only a few reports of epilepsy or seizures accompanying potassium abnormalities [9, 58, 59]. Nardone et al. [59] indicated that severe potassium abnormalities may provoke fatal arrhythmias or muscle paralysis before central nervous system symptoms appear. We might have encountered rare symptoms in the central nervous system in our case because we were able to appropriately decrease potassium concentrations, despite extremely severe hyperkalemia, without the occurrence of fatal arrhythmias or muscle paralysis. Furthermore, although previous reports indicate that more than one electrolyte disturbance can occasionally coexist in clinical settings [9, 58], our case had no other electrolyte disturbances except for potassium abnormality; this finding highlights the association between potassium abnormality and NCSE in this case.

In 2015, the ILAE proposed a new definition of status epilepticus, as follows: “Status epilepticus is a condition resulting either from the failure of the mechanisms responsible for seizure termination or from the initiation of mechanisms, which lead to abnormally, prolonged seizures (after time point t1). It is a condition that can have long-term consequences (after time point t2), including neuronal death, neuronal injury, and alteration of neuronal networks, depending on the type and duration of seizures” ([6] (p.1517). In semiology, forms of status epilepticus without prominent motor symptoms may be summarized as NCSE [6]. Notably, the frequency of situation-related NCSE is higher than might be expected in daily clinical settings [5, 46].

Hyperkalemia is potentially life-threatening, and the findings reported here do not suggest that potassium imbalances should not be rapidly corrected. Nevertheless, prompt EEG should be considered in patients with renal dysfunction or electrolyte disturbance who experience an acute state of confusion.

## Data Availability

The datasets used in this study are available from the corresponding author on reasonable request.

## Abbreviations

CKD: Chronic kidney disease
EEG: Electroencephalogram
GI: Glucose–insulin
HD: Hemodialysis
ILAE: International League Against Epilepsy
MeSH: Medical Subject Headings
NCSE: Non-convulsive status epilepticus
